# Machine learning reveals interhemispheric somatosensory coherence as indicator of anesthetic depth

**DOI:** 10.3389/fninf.2022.971231

**Published:** 2022-09-12

**Authors:** Dominik Schmidt, Gwendolyn English, Thomas C. Gent, Mehmet Fatih Yanik, Wolfger von der Behrens

**Affiliations:** ^1^Institute of Neuroinformatics, Department of Information Technology and Electrical Engineering (D-ITET), ETH Zurich, University of Zurich, Zurich, Switzerland; ^2^Neuroscience Center Zurich (ZNZ), Eidgenössische Technische Hochschule Zürich (ETH), University of Zurich, Zurich, Switzerland; ^3^Anaesthesiology Section, Vetsuisse Faculty, Department of Clinical Diagnostics and Services, University of Zurich, Zurich, Switzerland

**Keywords:** depth of anesthesia, gradient boosting, cortico-cortical coherence, mouse, somatosensory cortex (S1)

## Abstract

The goal of this study was to identify features in mouse electrocorticogram recordings that indicate the depth of anesthesia as approximated by the administered anesthetic dosage. Anesthetic depth in laboratory animals must be precisely monitored and controlled. However, for the most common lab species (mice) few indicators useful for monitoring anesthetic depth have been established. We used electrocorticogram recordings in mice, coupled with peripheral stimulation, in order to identify features of brain activity modulated by isoflurane anesthesia and explored their usefulness in monitoring anesthetic depth through machine learning techniques. Using a gradient boosting regressor framework we identified interhemispheric somatosensory coherence as the most informative and reliable electrocorticogram feature for determining anesthetic depth, yielding good generalization and performance over many subjects. Knowing that interhemispheric somatosensory coherence indicates the effectively administered isoflurane concentration is an important step for establishing better anesthetic monitoring protocols and closed-loop systems for animal surgeries.

## 1. Introduction

General anesthesia is commonly used in surgical procedures and acute experiments performed on laboratory animals in both fundamental and biomedical research. Exposure to general anesthetic agents strongly perturbs multiple brain networks and can have profound, lasting effects on the physiology of exposed animals (Franks, 2008; Bajwa et al., 2018; Pal et al., 2020). In order to minimize both the acute and chronic effects of anesthesia while also safeguarding the welfare of laboratory animals during surgery, the exposure to anesthetic agents should be expertly balanced. Namely, the administered anesthesia should be sufficient to maintain the animal in an unconscious state, while still minimally dosed to reduce the anesthetic's acute effect on brain function and its longitudinal effect on general physiology.

These anesthetic constraints are well-known in human medicine where, to prevent post-operative complications, general anesthesia should be titrated to avoid detrimental physiological effects (Eger et al., 1965; Dumont, 2012). To facilitate an anesthetic delivery that balances the demands of interoperative awareness and adverse effects, a significant amount of research has focused on measuring the human depth of anesthesia (DoA) (Nguyen-Ky et al., 2012; Ferdous and Kiber, 2014; Sadrawi et al., 2015). Such work has prominently led to the development of the proprietary Bispectral Index Score (BIS), which makes use of several electroencephalographic parameters to estimate DoA, and has been established as the predominant anesthetic monitor used during human surgeries (Dumont, 2012). Other published approaches for human DoA estimation rely on non-linear features extracted from electroencephalographic measurements or evoked potentials (Al-Kadi et al., 2013), which are used as inputs to traditional machine learning algorithms or artificial neural networks (Li et al., 2020; Abel et al., 2021).

While methods to estimate DoA in humans are well-developed and validated, for laboratory animals, and in particular mice, the most commonly used animal model in biomedical research, specific techniques and findings remain sparse (Hickman et al., 2017). Previous work has investigated closed-loop anesthetic delivery in rats to control the electroencephalogram-determined burst suppression, a signature of inactivated brain states (Ching et al., 2013; Yang et al., 2019). Another study has linked human DoA techniques to viable methods in neonatal mice using intracortical electrophysiology (Chini et al., 2019). More recent research has helped to elucidate the signatures of anesthesia induced effects on electrocorticogram recordings in rats (Wang et al., 2022). Despite prior work, a gap remains in the understanding of DoA monitoring in adult mice and of the specific features of electroencephalographic signals beyond burst suppression that are modulated by anesthesia. Further research has investigated alternative physiological measures for their usefulness in monitoring anesthetic depth, however heart rate and blood pressure have been shown to less accurately assess DoA than the bispectral index, suggesting that methods for monitoring anesthetic depth in mice based upon electroencephalographic signals may be most effective (Jaber et al., 2015).

In the present study, we want to address this lack of robust indicators for monitoring DoA in mice. We set out to identify features of brain activity that are modulated by anesthetic depth. While performing electrocorticogram recordings with concurrent sensory stimulation in mice, we systematically varied the anesthetic depth by changing the isoflurane concentration. Different features of the electrocorticograms were then provided to a machine learning framework to estimate the instantaneous anesthetic depth. Our findings elucidate that interhemispheric somatosensory coherence varies with the isoflurane anesthesia, that it has good predictive capacity and is less subject to interindividual variability. These properties of interhemispheric somatosensory coherence make it a useful feature for designing a future DoA monitoring systems in mouse models.

## 2. Results

**Figure 1:**
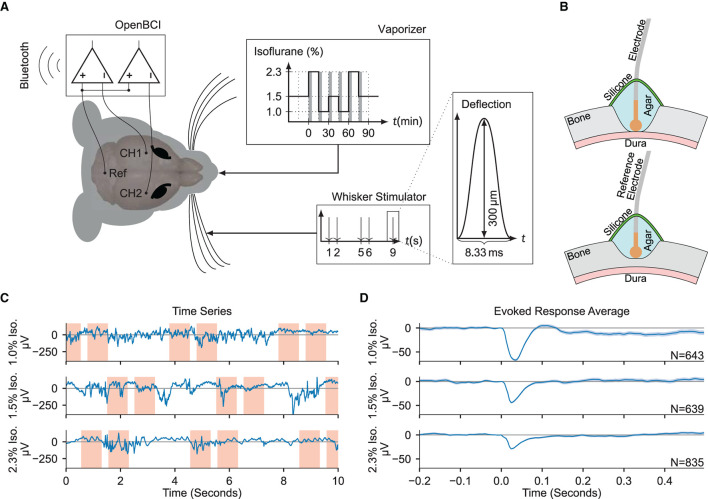
Electrocorticogram signals are extracted for estimation of anesthetic depth. **(A)** Schematic overview of the recording setup. Two electrodes over the somatosensory cortices (CH1 and CH2) were measured against a common reference over cerebellum (Ref), while stimulating the right whiskers and varying the isoflurane concentration. The gray shaded bars in the “Vaporizer” inset mark the first 5 min of each given isoflurane concentration segment which were excluded from statistical analysis. **(B)** (Top) Craniotomy over barrel cortices. The Ag/AgCl electrodes were placed directly on the dura, then covered in phosphate-buffered saline based agar and two component silicone. (Bottom) Partial craniotomy over cerebellum for the reference electrode, drilled to 20% thickness, and covered as above. **(C)** Example ECoG traces recorded using the OpenBCI during different administered isoflurane concentrations. Red shaded areas denote the [−0.2, 0.5 s] interval around a stimulus. **(D)** The evoked responses averaged over all trials in each isoflurane concentration block, zero-aligned at *t* = 0. The shaded area indicates the 2σ-range of the standard error of the mean.

Acute experiments were conducted on eleven adult female mice. Two epidural electrocorticogram (ECoG) electrodes were placed above the right- and left- hemisphere somatosensory cortices (see Section 4). Throughout the experiment, stimulation of the right whisker pad was used to evoke somatosensory responses, yielding the right- and left- hemispheres as the ipsi- and contra- lateral hemispheres to simulation, respectively. Concurrent to the ECoG recordings and somatosensory stimulation, animals were subjected to an anesthesia protocol that varied the concentration of administered isoflurane in consecutive blocks of approximately 15 minute duration (Figures 1A,B). Signal features were extracted from both ECoG channels, tested for anesthetic modulation, and ultimately provided as input to a gradient boosting regressor trained to estimate two proxies of anesthetic depth, namely the administered isoflurane concentration and the evoked response amplitude. The regressor was trained and evaluated across data collected from all experimental animals in a leave-one-out cross-validation scheme, showing good generalization errors across the entire population.

### 2.1. Electrocorticogram feature qualities

Statistical and spectral features were extracted from the ECoG signals and the effects of varying the administered isoflurane concentration were evaluated (Figures 1C,D). An exhaustive list of the tested features and their median values across all administered isoflurane concentrations, as well as the statistical significances of their anesthetic modulation, can be found in Supplementary Tables 1, 2. Similar to results in humans (Ching et al., 2012; Akeju et al., 2014; Liang et al., 2015), several features showed significant dependency on the administered isoflurane concentration, specifically: sample entropy (*p* = 5.35 × 10-5), interhemispheric somatosensory coherence (*p* = 4.08 × 10-5), Lempel-Ziv complexity (LZC) (*p* = 1.93 × 10-3), and the burst suppression ratio (*p* = 9.04 × 10-3).

**Figure 2:**
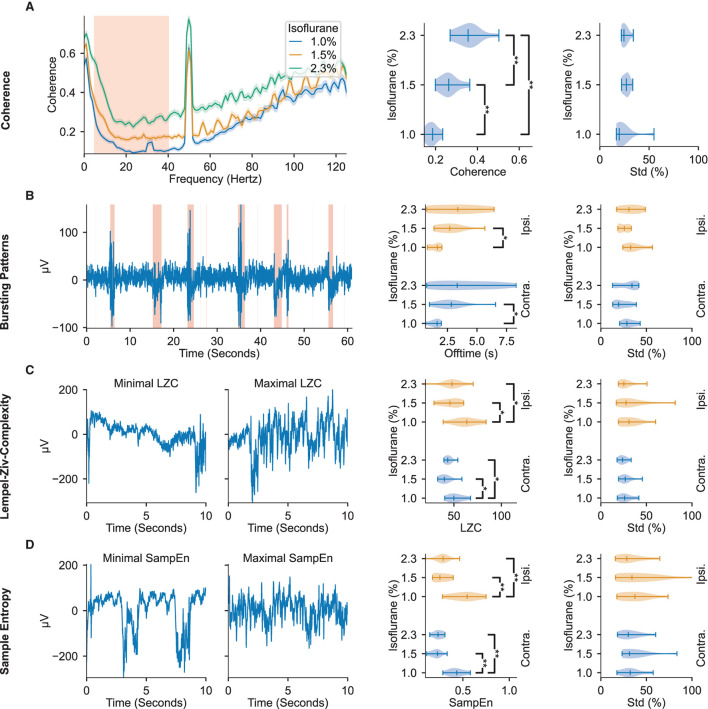
ECoG signal features display modulation to administered isoflurane concentrations. The leftmost column depicts representative examples of the features found to be most modulated by changes in administered isoflurane concentrations. The middle column depicts violin plots of the respective feature averages over animals across isoflurane concentrations. The rightmost column depicts violin plots of the respective standard deviation within the different isoflurane segments, relative to the mean value in the segment. All violin plots display the minimum, maximum, and median values of the distributions. The regions highlighted in red in the left panels depicts the data used in further analysis shown in the right panels. **(A)** Interhemispheric somatosensory coherence based on the unfiltered raw-data. The traces illustrate median and 95 % confidence interval calculated over all isoflurane blocks (right panels: 5–40 Hz). **(B)** Bursting Patterns. Shaded regions in representative example depict signal portions classified as burst. **(C)** Lempel-Ziv Complexity. Representative examples of signal segments with maximum and minimum LZC. **(D)** Sample Entropy. Representative examples of signal segments with maximum and minimum sample entropy. Significance testing computed with two-sided Mann–Whitney *U*-test across isoflurane concentrations with three Benjamini-Hochberg False Detection Rate controls (Benjamini and Hochberg, 1995). Significant results are denoted with ^*^p < 0.05, ^**^p < 0.01. Precise *p*-values are listed in Supplementary Table 1.

The interhemispheric somatosensory coherence between the two somatosensory cortex channels within the spectrum of 5 Hz to 40 Hz, comprising the theta, alpha, beta, and low gamma bands, significantly increases with administered isoflurane concentration (see Figure 2A). Important for DoA estimation, the interhemispheric somatosensory coherence exhibits highly significant differences across all tested isoflurane regimes, indicating a robust modulation across the spectrum of tested dosages.

As depicted in Figure 2B, increasing administered isoflurane concentration affects the burst suppression ratio by inducing more extended off (i.e., suppression) times. Increased offtime corresponding to increased administered isoflurane concentrations can be observed in both the contra- and ipsilateral hemispheres, though the former shows a higher statistical significance (*p* = 4.31 × 10-3). Notably, while the hemisphere contralateral to stimulation exhibits a significant dynamic between offtime and administered anesthetic concentration, the on (i.e., bursting) times are not significantly effected by the administered anesthetic concentration (see Supplementary Table 1), indicating that the offtime measurement may be a more effective indicator of DoA than the overall burst suppression ratio of on- to off- times.

Both LZC (i.e., the compressibility of the signal) and sample entropy values (i.e., the randomness of the signal) peak for the lowest administered isoflurane concentration of 1.0 %, and are significantly different from the values observed at the higher concentrations of 1.5 and 2.3 % (*p* ≤ 1.28 × 10-2 for LZC and *p* ≤ 2.91 × 10-3 for sample entropy, illustrated in Figures 2C,D). The increase of almost 100 % for sample entropy and 20 % to 30 % for LZC at lower anesthetic levels is observed in the electrode channels both contralateral and ipsilateral to the location of whisker stimulation (Figures 2C,D).

The distribution of all four of these features are appreciably separated across different administered isoflurane concentrations, rendering them useful measures for robust estimation of anesthetic depth. However, while the interhemispheric somatosensory coherence values clearly distinguish the three different isoflurane regimes, sample entropy, LZC, and the burst suppression ratio do not show significant differences between the two highest concentrations of administered isoflurane (1.5 and 2.3 %). Although the aforementioned features displayed consistent modulation by the administered isoflurane concentration, others we tested did not reliably vary with the anesthesia protocol (Supplementary Tables 1, 2). Aligned with prior research in rats (Antunes et al., 2003), modulation of the spectral edge frequency and 1/*f*-slope was not detected in our population of mice. We observed that the absolute power density reduces with increasing anesthesia (Supplementary Table 2), but is subject to large inter-animal variations (a standard deviation larger than 62 % of the mean), likely rendering these frequency related features ineffective in identifying consistent trends across animals without prior baseline knowledge.

### 2.2. Estimator performance

**Figure 3:**
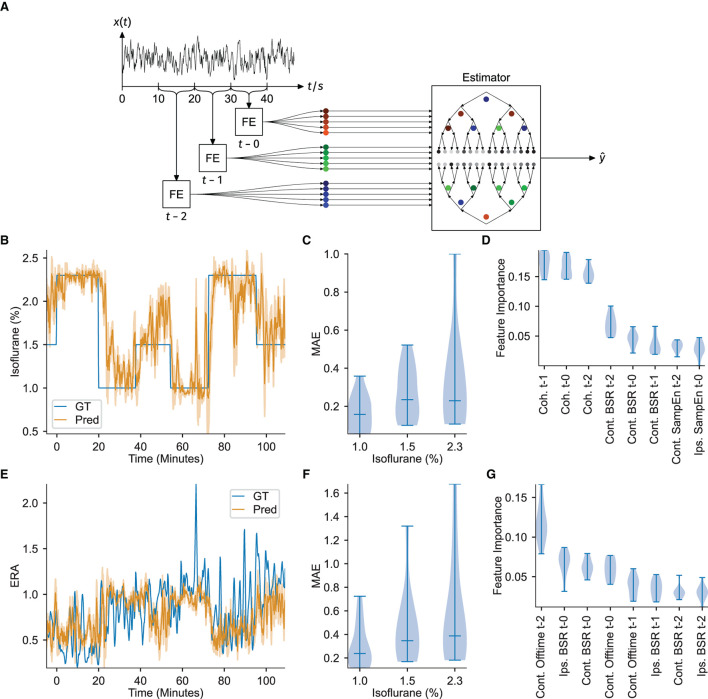
ECoG features serve as input to a gradient boosting regressor to successfully estimate DoA. Feature importances indicate interhemispheric somatosensory coherence as a critical DoA readout. **(A)** Overview of feature extraction and estimator workflow. Notch and high-pass filtered signal traces were extracted in 10 s blocks, signal features were calculated (FE), and then the three most recent consecutive blocks provided as input to estimate a target variable ŷ *via* a gradient boosting ensemble. **(B)** Example of the estimation of isoflurane concentration. GT denotes the measured ground-truth. The filled area under the predicted curve shows the standard error of the past minute. **(C)** Distribution of the Mean Absolute estimation Error (MAE) over all animals in each isoflurane regime, corresponding to estimation of the isoflurane concentration. **(D)** Feature importance over all folds. The interhemispheric somatosensory coherences are the most important features, over almost all folds, corresponding to estimation of the isoflurane concentration. **(E)** Example estimation of the evoked response attenuation (ERA). GT denotes the measured ground-truth. The filled area under the predicted curve shows the standard error of the past minute. **(F)** MAE over all folds or animals (*n* = 11), corresponding to estimation of the ERA. **(G)** Distribution of the feature importances over all animals, corresponding to estimation of the ERA. All the violin plots indicate the minimum, maximum and median values.

To further test the power of the ECoG features to estimate the instantaneous anesthetic depth, the extracted values were provided as input to a customized machine learning framework. A gradient boosting regressor with 100 boosting steps and maximum tree-depth of three was trained with the values of all of the previously described extracted features from the three most recent 10 s recording windows (*t-0*: 0 s to -10 s, *t-1*: -10 s to -20 s, *t-2*: -20 s to -30 s) as inputs and targeted two measures of anesthetic depth, namely, the administered isoflurane concentration and the evoked response amplitude (depicted in Figure 3A). Gradient boosting was chosen in a pre-trial review among Support Vector Regression (SVR) with Gaussian kernels, SVR with linear kernels, K-nearest neighbors and standard linear regression after exhibiting the best performance. Estimators for each anesthetic depth target were each trained eleven times, using a leave-one-out cross-validation scheme (i.e., for each iteration, one animal was removed from the whole dataset before training and the estimation performance evaluated against that animal). Individual feature importances (measured by the Gini gain, i.e., the total estimation improvement achieved by inclusion of the feature) were then extracted and their statistics over all 11-folds were considered. Stable estimation should yield similar feature importances over all folds. Such stability was observed when estimating the administered isoflurane concentration, however, estimation across folds was less reliable when the regressor was trained on evoked response amplitude, as shown in Figure 3G.

The administered isoflurane concentration was selected as the first target variable for DoA estimation, yielding estimators capable of inferring the effective isoflurane concentration. Similar to the concepts underlying the bispectral index score used in human surgeries, training the gradient boosting regressor to estimate the administered isoflurane concentration over a population of mice results in an estimate reflective of the ECoG feature values expressed by the average mouse at the specified isoflurane concentration. Furthermore, under certain assumptions, estimating the administered isoflurane concentration is similar to estimating the true depth of anesthesia (see Section 4 for mathematical derivations).

Estimation of the administered isoflurane concentration performed robustly over all animals [mean absolute error over folds: 0.238 ± 0.076 (mean ± std), *R*^2^-score: 0.576 ± 0.289]. Since the test set target variable is binned into three discrete isoflurane values, treating the isoflurane regression results as classification *via* nearest-neighbor quantization additionally allows for evaluation of standard multi-class classification metrics. Averaging over all One-vs.-All pairs yields an accuracy of 0.715 ± 0.134 (mean ± std), an *F*1-score of 0.699 ± 0.156, a precision of 0.780 ± 0.088 and recall of 0.714 ± 0.134. Figure 3B depicts the regression results over time for an example animal and demonstrates that the estimation quickly captures changes in administered isoflurane concentration. The stability of estimating this variable is further demonstrated by the low variance in estimation performance over all 11-folds, particularly for administered isoflurane concentrations of 1.0 and 1.5 %, depicted in Figure 3C. Figure 3D displays the feature importance extracted from all 11-folds. The high accuracy achieved for estimating the administered isoflurane concentration establishes our methodology is a proof-of-concept depth of anesthesia monitoring system for mice.

Analysis of the feature importances using the Gini gain shows that the interhemispheric somatosensory coherence between the two barrel cortex electrodes is critical for estimation, with the interhemispheric somatosensory coherence from the oldest time block (i.e., *t*−2, representing the window 30−20 s before current time, from Figure 3D) exhibiting the strongest importance for estimation. Sample entropy in the barrel cortex electrode located ipsilateral to the whisker stimulation was also important for estimation, again with the oldest values (*t*−2) displaying highest importance. Finally, for estimation of the administered isoflurane concentration, the burst suppression ratio for the ECoG channel contralateral to the whisker stimulation hemisphere demonstrated to be useful.

To rule out that the estimators use elapsed time as a hidden variable and subsequently learn a static map from elapsed time to isoflurane concentration, an identical gradient-boosting regressor was trained to estimate elapsed time. The performance-metrics of both estimators were then tested for correlation, yielding no significant correlation (see Supplementary Figures 4, 5).

Evoked response amplitude was selected as an alternative target variable for DoA estimation in order to test for properties of anesthetic depth systematically reflected in the neural response to peripheral stimulation. To perform this estimation, rather than using the raw maximum evoked response amplitude (as would be measured in volts), a unit-less ratio of the evoked response amplitude, as recorded from the ECoG channel contralateral to peripheral stimulation, was calculated. The unit-less ratio was computed using a moving-window average of the stimulus-by-stimulus maximum evoked response amplitude and dividing these averaged values across isoflurane concentration blocks by those computed during all 1.0 % isoflurane blocks. If the evoked response amplitude would scale similarly across varied anesthetic depths between animals, an accurate, stable estimation could be achieved.

Training the gradient boosting regressor to estimate the evoked response amplitude proved less accurate and robust [mean absolute error over folds: 0.446 ± 0.252 (mean ± std), *R*^2^-score: -1.103 ± 2.009, mean absolute errors depicted in Figure 3F]. While the estimation was accurate for several folds, or several animals, the results did not generalize well across all folds (example regression results for one fold depicted in Figure 3E). This irregularity can be observed in Figure 3G, which depicts highly variable feature importance.

## 3. Discussion

Here we have investigated the effect of varying concentrations of administered isoflurane anesthesia on electrocorticogram features and explored the capacity of these features to estimate the instantaneous DoA in mice. Our results show that many of the important features previously identified in human models translate also to mouse models, suggesting that elements of techniques developed for monitoring of human DoA may further warrant incorporation into systems targeting laboratory animals. Our analysis confirms the modulation of interhemispheric somatosensory coherence (Michelson and Kozai, 2018), burst suppression ratio (Tonner and Bein, 2006), Lempel-Ziv complexity, and sample entropy (Liang et al., 2015) through administered isoflurane concentration. However, we observed a number of features that do not appear to be significantly effected by anesthesia. Notably, the spectral measures of 1/*f*-slope (Antunes et al., 2003; Barter et al., 2005), spectral edge frequencies and absolute power distributions either show no modulation or high inter-animal variability, rendering them unsuitable for estimation.

Isoflurane is one of the most commonly used inhalable anesthetic agents for laboratory animals and has an inhibitory effect on excitatory neurons via a number of molecular mechanisms (de Sousa et al., 2000; Franks, 2008). This reduction in excitation results in a reduced stimulation of inhibitory interneurons which later causes a depletion of endogenous inhibition (Ferron et al., 2009). Such cycles of increased and reduced inhibition are manifest in the burst suppression ratio, where dose-dependent isoflurane delivery modulates the ratio of on- and off-times in mice (Brown et al., 2018). Our experiments also reveal a dependence between administered isoflurane concentrations and the burst suppression ratio, however the computed ratio values exhibit significant differences only between the lowest administered isoflurane concentration and the higher two concentrations (e.g., 1 vs. 1.5 and 2.3 %). Our data indicates that the burst suppression ratio is not a strong indicator of anesthetic depth at higher DoA. The mechanisms of isoflurane that contribute to the burst suppression ratio similarly impact the Lempel Ziv Complexity and Sample Entropy features, where induced extended offtimes create a more stable, compressible signal. Similar to our observation in the burst suppression ratio, both of these complexity features exhibit stronger significance between 1 vs. 1.5 and 2.3 %, again yielding their values less useful in distinguishing between higher levels of anesthetic depth.

As indicated by the feature importances in Figure 3D, interhemispheric somatosensory coherence is a robust measure for estimating depth of anesthesia, also between higher concentrations of administered isoflurane. The variation observed in interhemispheric somatosensory coherence from 5 to 40 Hz, comprising the theta, alpha, beta, and low gamma bands, across different anesthetic depths has, to the best of our knowledge, not been reported in recordings made from the somatosensory cortices. Previous reports in humans and rats have identified modulation of alpha coherence in recordings made from somatosensory and frontal cortices under propofol anesthesia (Cimenser et al., 2011; Supp et al., 2011; Baker et al., 2014). Further studies using isoflurane in rats have indicated that coherence between primary motor and visual cortices during peripheral sensory stimulation declined as delivered anesthetic concentrations increased (Imas et al., 2006). Our results in recordings made from both hemispheres of the somatosensory cortex may reflect that increased isoflurane administration enforces more phase-coherence between thalamocortical projections to the two sensory hemispheres (Ching et al., 2010). While our experiments cannot reveal the precise mechanisms of this isoflurane induced coherence, the resulting effect proves to be a critical component for estimating DoA across all depths.

Using all of the aforementioned features as input to the gradient boosting regressor yielded poor performance when the estimation of the evoked response amplitude was targeted. This poor performance could be explained by the intra- and inter-animal variability observed in the somatosensory evoked response. Previous research investigating the effects of anesthesia in mouse models identified that the average amplitudes of visual evoked potentials were not significantly affected by variations in administered isoflurane concentration and that evoked responses exhibited large trial-by-trial variability within delivered concentration blocks (Aggarwal et al., 2019). Our results, coupled with these previous findings, indicate that evoked responses recorded from primary sensory cortices across sensory modalities may not be useful readouts for DoA.

The administered isoflurane concentration estimator performs reliably over all animals as shown in Figures 3B,C. All of the identified features and estimators can be evaluated with a low latency of 10 s. This evaluation latency time makes the use of the identified features suitable for integration into closed-loop anesthetic delivery (CLAD) systems (Ching et al., 2013; Yang et al., 2019), which could target specific variables reflective of the DoA. Further enhancements could be made to estimator performance *via* a more exhaustive exploration of possible signal features, such as the bispectral coherence (Li et al., 2012), or by the implementation of neural networks to replace manual feature extraction (Sadrawi et al., 2015; Li et al., 2020).

The use of the identified electrocorticogram signal features exhibiting modulation by anesthesia in DoA monitoring systems for mice could provide numerous improvements to animal welfare and biomedical research, ultimately allowing for more precise experimental control and informed adjustment of the administered anesthesia. A potential future scenario would be an (clinical) anesthesia monitoring systems that continuously measures the effectively administered isoflurane anesthesia that is independent of the individual subject. This may be particular useful in scenarios where the drug dosage administration is not well controlled or prone to miscalculation. However, such a monitoring system certainly can go beyond and predict the actual anesthetic depth when validated with other physiological parameters. The development of such a monitoring system would require testing the suitability of the identified ECoG signal features obtained through less invasive electroencephalography (EEG) recording methods and testing whether these results generalize to different experimental paradigms (e.g., without whisker stimuli or using a different anesthetic agent). Further, we validated our system using isoflurane anesthesia, the most common and recommended modality for acute recordings and recovery procedures in experimental mice (Gargiulo et al., 2012). A valuable next step would be to extend this to other anesthetic regimens, a finding which would not only allow standardization of anesthetic depth across experiments, but also shed light upon the neuronal mechanisms involved in anesthetic induced unconsciousness (Franks, 2008). Finally, a translation of our results into a clinical system would require to test if interhemispheric somatosensory coherence is a predictive DoA feature for different pathological conditions as well. Such a validation and new training of the network may be, in particular, necessary for brain disorders which are known to affect the electrocorticogram and EEG including neurodegenerative (e.g., in different mouse models of Alzheimer's diseases; Kent et al., 2018), neurological (e.g., after striatal stroke in mice; Baumann et al., 2006), and neuropsychiatric conditions (e.g., SAPAP3^-/-^ mice which are a model for anxiety and obsessive compulsive disorders; Lei et al., 2019).

## 4. Methods

### 4.1. Animal experiments

In this study, eleven adult female C57BL/6J mice (age 98.0 ±19.3 days, weight 21.00 ±1.83 g), housed in enriched cages of four mice in husbandry facilities with a 12-h inverted light/dark cycle, supplied by Charles River Laboratories were used for acute experiments. All experimental and surgical procedures were approved by the local veterinary authorities and corresponding ethics committees of the Canton Zurich, Switzerland, and were carried out in accordance with the guidelines published in the European Communities Council Directive of November 24, 1986 (86/609/EEC). This study is reported in accordance with ARRIVE guidelines.

Mice were briefly induced with 3 % isoflurane in oxygen anesthesia and injected with 1 mg/kg Meloxicam (Boehringer Ingelheim, Ingelheim am Rhein, Germany) as analgesic. Remaining surgical procedures were completed under 2 % isoflurane in oxygen supplied at a 1 L/s flow rate. Body temperature was regulated at 37 °C with a homeothermic blanket control unit (Harvard Apparatus, Holliston, Massachusetts). Animals were mounted in a stereotactic frame and the scalp was removed to expose the skull.

Three silver electrodes were then positioned onto the skull and affixed using dental cement (Dentsply Sirona, York, Pennsylvania). Electrodes were manufactured with 250 μm thin Teflon (PTFE) coated silver wire (Goodfellow Cambridge Limited, Huntingdon, England). Teflon coating was removed to expose the wire end, which was subsequently molten to a sphere (diameter 500 μm) and chlorided to improve electrochemical stability (Geddes et al., 1969).

Two craniotomies over the barrel field of primary somatosensory cortex of both hemispheres (3.5 mm lateral and 1.5 mm caudal of bregma) were performed to expose an 1 mm diameter circular region of intact dura. A third partial craniotomy was drilled over the cerebellar region (2 mm lateral and caudal of lambda) until 80 % of the skull was removed. All three rounded electrode tips were then placed into the craniotomies, with both barrel cortex electrodes making contact with the dura and the reference electrode contacting the thinned skull above cerebellum. Craniotomies and electrode tips were covered with a phosphate buffered saline agar (PBS) mixture (2 % PBS) (Sigma-Aldrich, St. Louis, Missouri). The agar was subsequently covered with a two-component silicone (World Precision Instruments, Sarasota, Florida) to prevent drying of the electrode sites. Silicone deposits over each craniotomy were isolated from one another to avoid electrical connectivity between recording sites. The skull surface was finally rinsed with de-ionized water to prevent parallel resistances that could interfere with the individual biological signals. Electrode impedances were typically around 10 k℧ to 20 k℧ and usually increased slightly during the recordings.

Multiple whiskers from the right hemisphere vibrissal field (rows B-D, arcs 1-3) were then secured around a glass capillary that was then positioned immediately tangential to the whisker pads. The capillary was affixed to a piezo-bending actuator (Piezo Systems, Woburn, Massachusetts) driven by a controller with a maximum output voltage of 150 V (Thorlabs, Newton, New Jersey). Whisker stimulation sequences were generated using custom LabVIEW code (National Instruments, Austin, Texas), which produced an analog waveform with a sample rate of 200 kHz and a resolution of 16 bits. The mechanical waveforms at the capillary tip were single 120 Hz raised cosine pulses (8.3 ms duration) with an amplitude of 300 μm (1.72°) and a peak velocity of 113.1 mm s-1 (648.8 ° s-1) (Musall et al., 2017), confirmed using a 0.1 μm resolution laser displacement sensor (Micro-Epsilon, Ortenburg, Germany). Throughout the recording, whiskers were repeatedly deflected with a 1 Hz train containing 2 s stimulus-on and stimulus-off periods (Figure 1A). Four of the eleven mice received tail pinches delivered *via* Hoffman clamp to produce data used in subsequent experiments. All time periods containing tail pinch stimulation were removed from the analyzed dataset.

Contemporaneous with the whisker stimulation and electrocorticogram recording, an anesthesia protocol was applied to achieve stable conditions at a variety of isoflurane concentrations. The protocol consisted of 15 min segments of isoflurane concentration percentages delivered in the following sequence: starting from 1.5%, then transitioning to the following segments: 2.3, 1.0, 1.5, 1.0, 2.3, and 1.5%. Isoflurane concentrations were selected to span a broad range of depths while remaining below dosages of 1.5 minimum alveolar concentrations as determined in mice (Cesarovic et al., 2010) and represent values of inhalable isoflurane recommended for maintenance of an anesthetized state in mice (Gargiulo et al., 2012). We used the inhalable isoflurane anesthesia, as it allows maximum temporal control of anesthetic depth over other injectable alternatives (Tremoleda et al., 2012). From each 15 min segment of a given isoflurane concentration, the first 5 min were excluded from the statistical analysis, yielding from each animal a dataset of six segments of 10 min duration (see gray bars in the inset “Vaporizer” of Figure 1A).

The completed setup was then enclosed in grounded aluminum foil for improved shielding against electromagnetic influences (e.g., from the proximal piezoelectric stimulators). Recordings were made using an open-source neural data acquisition platform (OpenBCI, Brooklyn, New York) with a gain of 24x and sample rate of 250 Hz. For all subsequent analysis, the gain factor was removed from the raw data, consequently all data reported is input referred. Electrode signals and whisker stimulation onsets were communicated to the recording computer *via* Bluetooth connection.

Data collected from all animals was considered to be in the experimental group. In order to best capture the unique inter-animal dynamics induced by the dosage of isoflurane anesthesia, data collected from all animals was a priori selected for inclusion in analysis.

### 4.2. Feature extraction

The recording setup yielded two electrocorticogram channels obtained from the barrel cortex electrodes located both ipsilateral and contralateral to the hemisphere of whisker stimulation. Signal traces were preprocessed using a forward-backward notch filter to remove line noise at 50 Hz with a quality factor *Q* = 30 and a forward-backward first order Butterworth high-pass filter above 0.1 Hz to eliminate signal drift. Both raw ECoG signals were extracted from each 15min isoflurane concentration block. The first 5 min of each block were excluded from analysis such as to ignore the transient effects of the previous concentration block. The following spectral and time domain features of interest were then extracted from consecutive, non-overlapping 10 s sequences from each signal block.

Spectral features extracted from both electrophysiology channels included the spectral edge frequency below which 95 % of the spectral power was contained (SEF 95) and the 1/*f*-slope fit over the frequency range 20 Hz to 40 Hz. Additionally, the interhemispheric somatosensory coherence was calculated between the two electrophysiology channels and averaged over the frequency range 5 Hz to 40 Hz. Finally, spectral power content in multiple EEG bands (Newson and Thiagarajan, 2019) were extracted, namely in the δ (0.1 Hz to 4 Hz), θ (4 Hz to 8 Hz), α (8 Hz to 13 Hz), β (13 Hz to 30 Hz), and γ (above 30 Hz) bands. All spectral features are based on Welch's power spectral density estimation calculated independently over each 10 s window.

To complement the spectral features, a variety of time domain features were identified for further use in the algorithmic determination of anesthetic depth. The sample entropy was computed over signal subsequences of 80 ms length. As a measure of compressibility of the electrophysiology channels, a binary sequence was extracted from the voltage traces by thresholding them against the median value in every signal block, and its Lempel-Ziv complexity calculated. The burst suppression ratio was calculated over the entire isoflurane concentration block to quantify the ratio of periods of high and low signal activity.

The described features were tested for modulation by isoflurane through averaging them over segments of equal isoflurane concentration for each mouse. The significance of this modulation was quantified by executing a Mann–Whitney *U*-Test for every pair of unequal isoflurane concentrations for each feature. To correct for multiple comparisons, a Benjamini-Hochberg False Detection Rate control (Benjamini and Hochberg, 1995) has been applied. Further details on feature calculations can be found in Supplementary material.

### 4.3. Estimation

As anesthetic depth is a value on a continuous spectrum, we perform a regression analysis to identify the current anesthetic state of the animal based on the extracted features. All extracted features were provided as inputs to a gradient boosting regressor implemented by the python scikit-learn package (Pedregosa et al., 2011). Such gradient boosting algorithms combine weak estimators *g*_*m*_ into a strong estimator *G*_*m*_ *via* superposition:


(1) $$\begin{array}{l} G_{M}\left(x\right)=\overset{M}{\underset{m=1}{\sum }}w_{m}g_{m}\left(x\right) \end{array}$$

with **w**∈ℝ^*m*^ weighting the individual estimators. Here the individual estimators are chosen to be shallow decision trees (with a maximum depth of three).

A decision tree is a binary tree whose leaf nodes are assigned a real valued number—the estimation. On every non-leaf node, a decision is made what child node to traverse to, based on a single feature and a threshold for that feature. Estimation using decision trees is thus a simple tree traversal, returning the value assigned to the leaf. In the training process, the optimal feature and threshold is determined for every node by iterating through all possible features, determining the optimal threshold (which can be done computationally efficient), and greedily choosing the feature and threshold that minimizes the error (Hastie et al., 2009). The decrease in error after the split based on this feature is tracked globally, which (summed over all decision trees and normalized over all features) is represented by the Gini gain computed in feature importance calculations (Breiman et al., 1983). In gradient boosting regression, every successive estimator is trained on the residual error of the superposition of all previous estimators, resulting in successively improved overall loss (Hastie et al., 2009).

Two target variables for the estimators were used, namely the administered isoflurane concentration and the evoked response amplitude. As input, the estimator was provided the values of each feature *x* in the set of all extracted features **x** described above at three consecutive 10 s sub-sequences, yielding a dataset of


(2) $$\begin{array}{l} D=\;\{\left([x_{0},x_{1},x_{2}],y_{2}),...,([x_{N-2},x_{N-1},x_{N}],y_{N}\right)\}\; \end{array}$$

where **x**_*n*_ represents the feature vector at the *n*-th 10 s sub-sequence, and *y* represents the anesthetic depth, given as either the administered isoflurane concentration of evoked response amplitude, in the same sub-sequence. For analysis, the datasets of all *n* = 11 mice were typically concatenated into a single dataset.

Using the datasets acquired from the *n* = 11 mice, leave-one-out cross-validation was performed, with each fold excluding the dataset recorded from one animal. The estimator was trained on the remaining *n* = 10 datasets, and evaluated on the excluded test animal, yielding an estimation of the generalization error. The training and evaluation set for the estimator for the *i*-th fold is thus:


(3) $$\begin{array}{ll} D_{\text{eval},i} & =\left[\begin{array}{cccc} {\text{x}}_{i,0} & {\text{x}}_{i,1} & {\text{x}}_{i,2} & y_{i,2}\\ {\text{x}}_{i,1} & {\text{x}}_{i,2} & {\text{x}}_{i,3} & y_{i,3}\\ \vdots & \vdots & \vdots & \vdots \\ {\text{x}}_{i,N_{i}-2} & {\text{x}}_{i,N_{i}-1} & {\text{x}}_{i,N_{i}}, & y_{i,N_{i}} \end{array}\right] \end{array}$$

(4) $$\begin{array}{ll} D_{\text{train},i} & =\left[\begin{array}{cccc} {\text{x}}_{0,0} & {\text{x}}_{0,1} & {\text{x}}_{0,2} & y_{0,2}\\ {\text{x}}_{0,1} & {\text{x}}_{0,2} & {\text{x}}_{0,3} & y_{0,3}\\ \vdots & \vdots & \vdots & \vdots \\ {\text{x}}_{0,N_{0}-2} & {\text{x}}_{0,N_{0}-1} & {\text{x}}_{0,N_{0}}, & y_{0,N_{0}}\\ {\text{x}}_{1,0} & {\text{x}}_{1,1} & {\text{x}}_{1,2} & y_{1,2}\\ \vdots & \vdots & \vdots & \vdots \\ {\text{x}}_{1,N_{1}-2} & {\text{x}}_{1,N_{1}-1} & {\text{x}}_{1,N_{1}}, & y_{1,N_{1}}\\ \vdots & \vdots & \vdots & \vdots \\ {\text{x}}_{M,N_{M}-2} & {\text{x}}_{M,N_{M}-1} & {\text{x}}_{M,N_{M}}, & y_{M,N_{M}} \end{array}\right]\backslash D_{\text{eval},i} \end{array}$$

**x**_*m, n*_ denotes the feature vector of the *n*-th block from the *m*-th mouse, *y*_*m, n*_ likewise the target variable on block *n* of mouse *m*. *N*_*m*_ denotes the number of 10 s blocks in the recording of mouse *m*.

While the administered isoflurane concentration was binned into the discrete values 1.0,1.5,2.3 in our experimental protocol, a regression approach was nevertheless preferred to classification, in order to capture the continuous nature of anesthetic depth and to better gauge the promptness of response without delays due to the quantization inherent to classification. Therefore, for estimation of the administered isoflurane, a surrogate classification metric has been used, by binning the estimated effective isoflurane concentration into the three bins 1.0, 1.5, 2.3 *via* nearest-neighbor quantization and calculating the 3-class classification scores with averaging over all One-vs.-All pairs.

To rule out that the regression framework is merely estimating the elapsed time as a hidden variable, and from that a linear map to the isoflurane curve, the following approach has been employed: an identical estimator is trained not on isoflurane or Evoked Response Attenuation (ERA), but on the elapsed time as ground-truth. Its *R*^2^-Score is then correlated using Spearmans R, to see whether good performance on time-estimation will also yield good estimation of isoflurane or ERA.

With this approach an estimator is established, capable of making anesthetic depth estimations by requiring a total of only 30 s of feature data for estimation, with a minimal latency of 10 s.

#### 4.3.1. Estimation for true depth of anesthesia

Estimating the administered isoflurane concentration can be understood as an anesthetic depth estimation similar to the bispectral index score. A population of mice has an average reaction to any given isoflurane concentration. Training over enough data yields an estimator that assigns the most probable isoflurane concentration at which the average mouse (over the training population) would have the observed reaction. We can further show that, with certain assumptions, estimating isoflurane concentration is equivalent to estimating the true depth of anesthesia. We do this by modeling the DoA problem as a Bayesian network, with probability density function $f\left(\vec{F},d,c\right)=f\left(\vec{F}|d\right)\cdot f\left(d|c\right)\cdot f\left(c\right)$ where $\vec{F}$ is the extracted features, *d* the true depth of anesthesia (which is a hidden variable), and *c* the administered isoflurane concentration.

An optimal estimator (minimizing a square loss), which has perfect knowledge of the density $f\left(\vec{F},d,c\right)$, will estimate $𝔼\left[d|\vec{F}\right]$ (Hastie et al., 2009). Since *d* is a hidden variable, we have no way to train on it, and thus settle on estimating $𝔼\left[c|\vec{F}\right]$. How does this surrogate compare to the optimal estimation?


(5) $$\begin{array}{ll} 𝔼\left[c|\vec{F}\right] & =\underset{c}{\int }c\cdot f\left(c|\vec{F}\right)dc \end{array}$$

This can be expanded if we marginalize over the hidden variable *d*, and by applying the Bayes rule we get:


(6) $$\begin{array}{l} =\underset{c}{\int }\underset{d}{\int }c\cdot f\left(c|d\right)f\left(d|\vec{F}\right)dddc \end{array}$$

Swapping the integrals results in an inner expectation:


(7) $$\begin{array}{l} =\underset{d}{\int }{𝔼}_{c|d}\left[c|d\right]\cdot f\left(d|\vec{F}\right)dd \end{array}$$

(8) $$\begin{array}{l} ={𝔼}_{d|\vec{F}}\;{𝔼}_{c|d}\left[c|d\right]\;\vec{F}\; \end{array}$$

We can see that what separates this estimator from the optimal one is a transformation from anesthetic depth to the average isoflurane concentration *h*:*d*↦*c*, *h*(*d*) = *E*[*c*|*d*]. Assuming that *h* is sufficiently linear where $f\left(d|\vec{F}\right)$ has most of its support, we can apply the linearity of the expectation and get the following approximation:


(9) $$\begin{array}{l} ={𝔼}_{d|\vec{F}}\;h\left(d\right)\;\vec{F}\; \end{array}$$

(10) $$\begin{array}{l} \approx h\;{𝔼}_{d|\vec{F}}\;d|\vec{F}\;\; \end{array}$$

It is thus approximately a linear transformation of the optimal estimation.

We estimate that the local linearity of *E*[*c*|*d*] should be fulfilled for reasonable values of *d*, i.e., between very high anesthetic depths and awake state.

## Data availability statement

The raw data supporting the conclusions of this article will be made available by the authors, without undue reservation.

## Ethics statement

The animal study was reviewed and approved by Veterinary office of the Canton of Zurich.

## Author contributions

GE and WB conceived the study. GE and DS designed the experiments, recording techniques, and wrote the manuscript with review from WB, MFY, and TCG. DS conceived of and conducted all data analysis. GE, TCG, and DS performed the experiments. All authors contributed to the article and approved the submitted version.

## Funding

This work was funded by the Swiss National Science Foundation (Project Grant No. 310030_172962). TCG was supported by the University of Zürich Forschungskredit (FK-017-64) and the Federal Food Safety and Veterinary Office of Switzerland (2.20.02). Open access funding provided by ETH Zurich.

## Conflict of interest

The authors declare that the research was conducted in the absence of any commercial or financial relationships that could be construed as a potential conflict of interest.

## Publisher's note

All claims expressed in this article are solely those of the authors and do not necessarily represent those of their affiliated organizations, or those of the publisher, the editors and the reviewers. Any product that may be evaluated in this article, or claim that may be made by its manufacturer, is not guaranteed or endorsed by the publisher.
