# Supplementary material for: Machine learning reveals interhemispheric somatosensory coherence as indicator of anesthetic depth
Source: Front Neuroinform. 2022 Sep 12;16:971231. doi: 10.3389/fninf.2022.971231 (PMC9510780; doi:10.3389/fninf.2022.971231)
Supplement: Supplementary file 1 [file Data_Sheet_1.pdf]

Supplementary Material

1 SUPPLEMENTARY DATA

1.1 Feature Extraction

For determining the burst suppression ratio, the detection of bursts is completed via simple thresholding of the high-pass filtered signal ($0.1\,\mathrm{Hz}$ first order Butterworth forward-backward high-pass). The threshold-value λ is calculated by taking the minimum of the local standard deviation of the signal. The local standard deviation of the signal is calculated by first low-pass filtering the high-pass filtered data y_{HP} with H to receive the local mean $m[n] = (H * y_{HP})[n]$, and then low-pass filtering the square distance from that mean, $\sigma^2[n] = (y_{HP}[n] - m[n])^2 * H[n]$. The threshold is hence $\lambda = \min_n \sqrt{|\sigma^2[n]|}$, with H a forward-backward first order Butterworth low-pass at $300\,\mathrm{Hz}$. This results in the binary sequence $b[n] = y_{HP}[n] < -\lambda$. The burst suppression ratio is then calculated as the local average of this sequence, i.e. BSR$[n] = b * G$ with G a forward-backward first order Butterworth low-pass at $120\,\mathrm{Hz}$. The on- and off-times are easily extracted directly from the binary sequence. We observed that mostly peak values change with isoflurane, hence an envelope filter was applied over the on- and off-times. This *non-linear* filter is defined as:

$$E[n] = \max\left\{y[n],\ y[n]b[0] + y[n-1]b[1] - a[1]E[n-1]\right\} \tag{S1}$$

With $a[0] = 1, a[1], b[0], b[1]$ the coefficients of a standard first order Butterworth low-pass filter at $100\,\mathrm{Hz}$. Unlike all other features, the bursting pattern is thus calculated continuously for the whole recording, and not in $10\,\mathrm{s}$ signal subsequences.

The *Lempel-Ziv Complexity* (LZC) is a measure of the compressibility of a signal. Each $10\,\mathrm{second}$ signal sequence was first thresholded against the median value, yielding a binary sequence which was then compressed using the LZC algorithm. The LZC value is then computed as the ratio of compressed to uncompressed binary sequence length Hu et al. (2006).

The *Sample Entropy* is a measure of complexity of subsequences of a time-series signal Richman and Moorman (2000). Similar signal subsequences of length $80\,\mathrm{ms}$ were identified, then the signal values at the next time-step were compared to yield the sample entropy value.

The *Evoked Response Amplitude* is extracted by evaluating the evoked responses in the interval $[0\,\mathrm{s}, 0.2\,\mathrm{s}]$, applying a moving window average (20 presentations in the past, 20 in the future) over the presentations, and extracting the minimal value in that interval. A representative unit-less ratio is calculated by dividing these instantaneous values against their average under $1.0\,\%$ isoflurane.

The *Spectral Edge Frequency* is a measure of power distribution. The spectral edge frequency was calculated to identify the frequency below which $95\,\%$ of the spectral power is contained.

The $1/f$-*slope* is the α-parameter of a curve-fit of the power spectral density to a function $\hat{S}_x x(f) = \frac{a}{f^\alpha}$. This has been done for the frequency range of $20\,\mathrm{Hz}$ to $40\,\mathrm{Hz}$ by first transforming the power spectral density into a log-log-space, and then via least-squares line fit.

The *Coherence* feature measures the correlation of two signals over frequency regions.The coherence between the two barrel cortex electrodes was calculated and the value averaged over $5\,\mathrm{Hz}$ to $40\,\mathrm{Hz}$.

2 SUPPLEMENTARY TABLES AND FIGURES

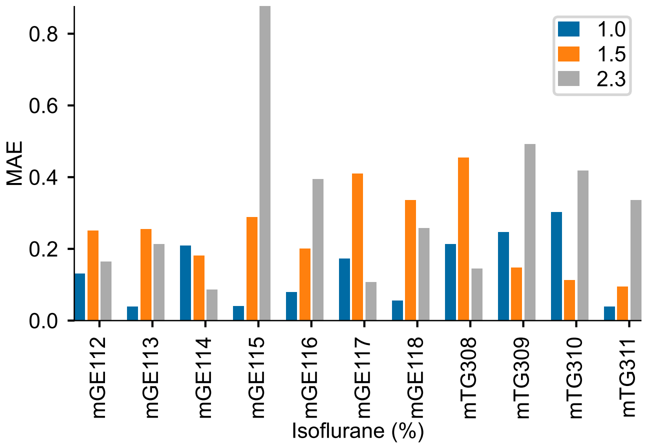

Figure S1. Minimum Absolute Error (MAE) of the administered isoflurane concentration estimation of each mouse.

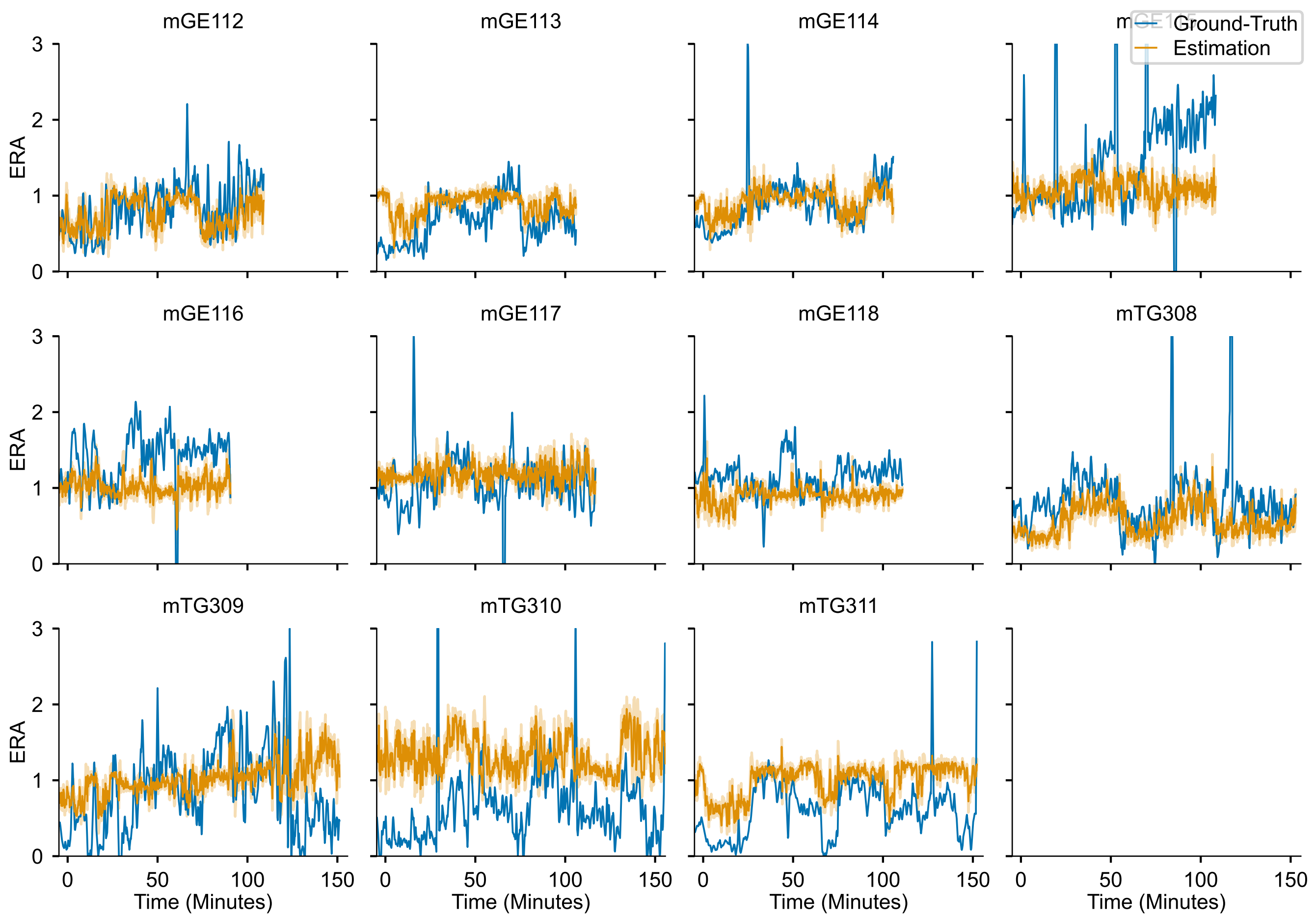

Figure S2. The evoked response amplitude estimation results of all the 11 folds. The traces shown are always evaluated on a dataset not seen in the training data. The shaded area denotes the standard error of the past minute

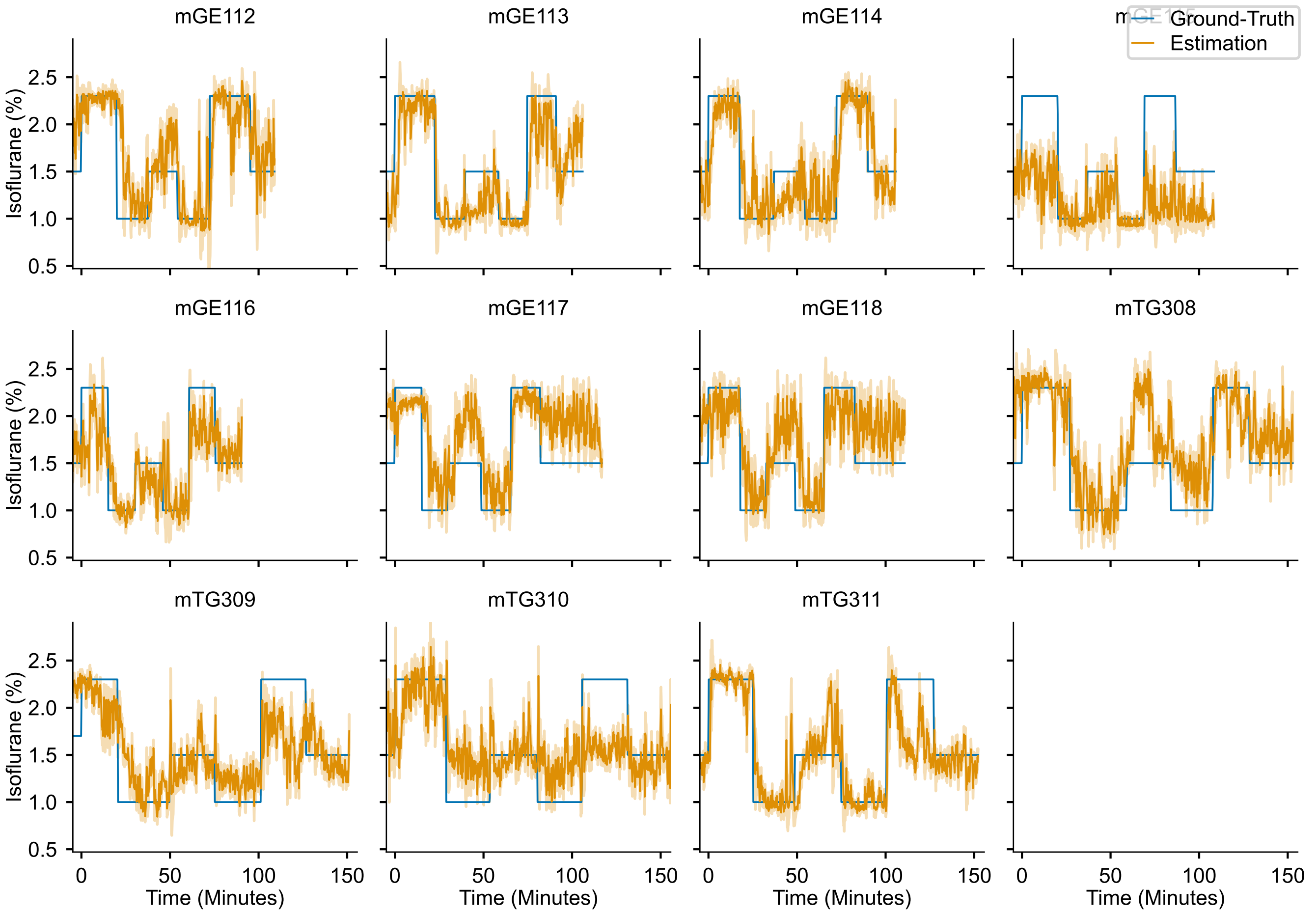

Figure S3. The administered isoflurane concentration estimation results of all 11 folds. The traces shown are always evaluated on a dataset not seen in the training data. The shaded area denotes the standard error of the past minute

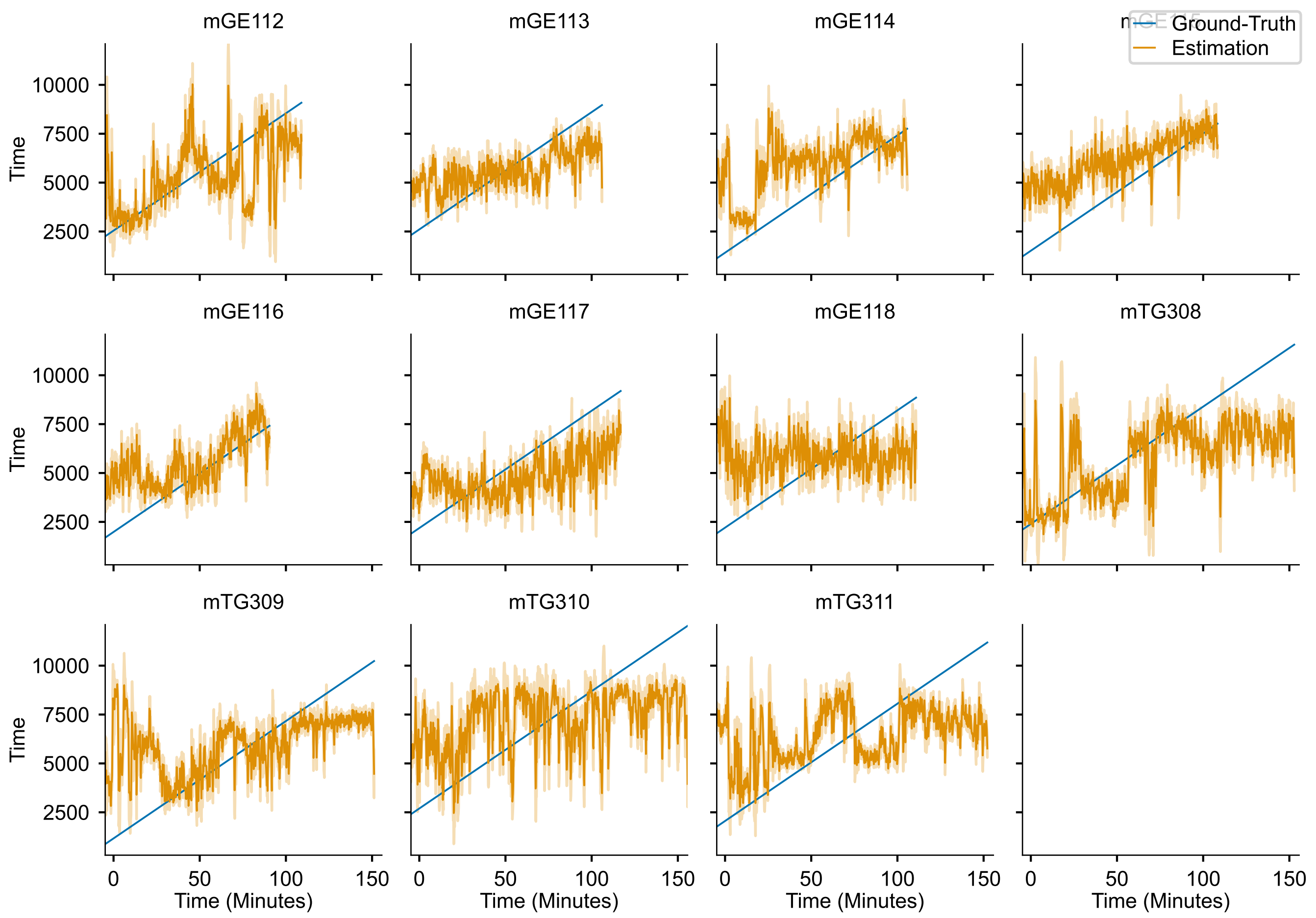

Figure S4. The elapsed time estimation results of all 11 folds. The traces shown are always evaluated on a dataset not seen in the training data. The shaded area denotes the standard error of the past minute

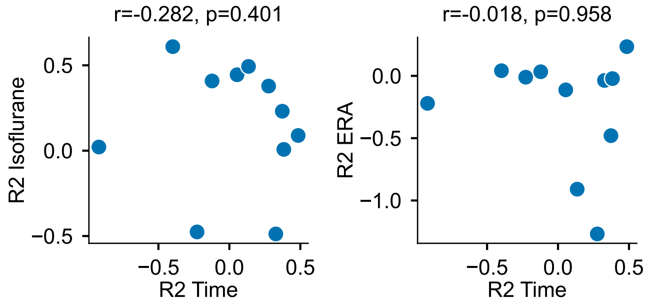

Figure S5. The Spearmans R Correlation coefficient and p-value of the R^2 performance score of isoflurane and Evoked Response Attenuation (ERA) estimation with time estimation. The performances are decorrelated, hence the estimators are unlikely to learn time as hidden variable

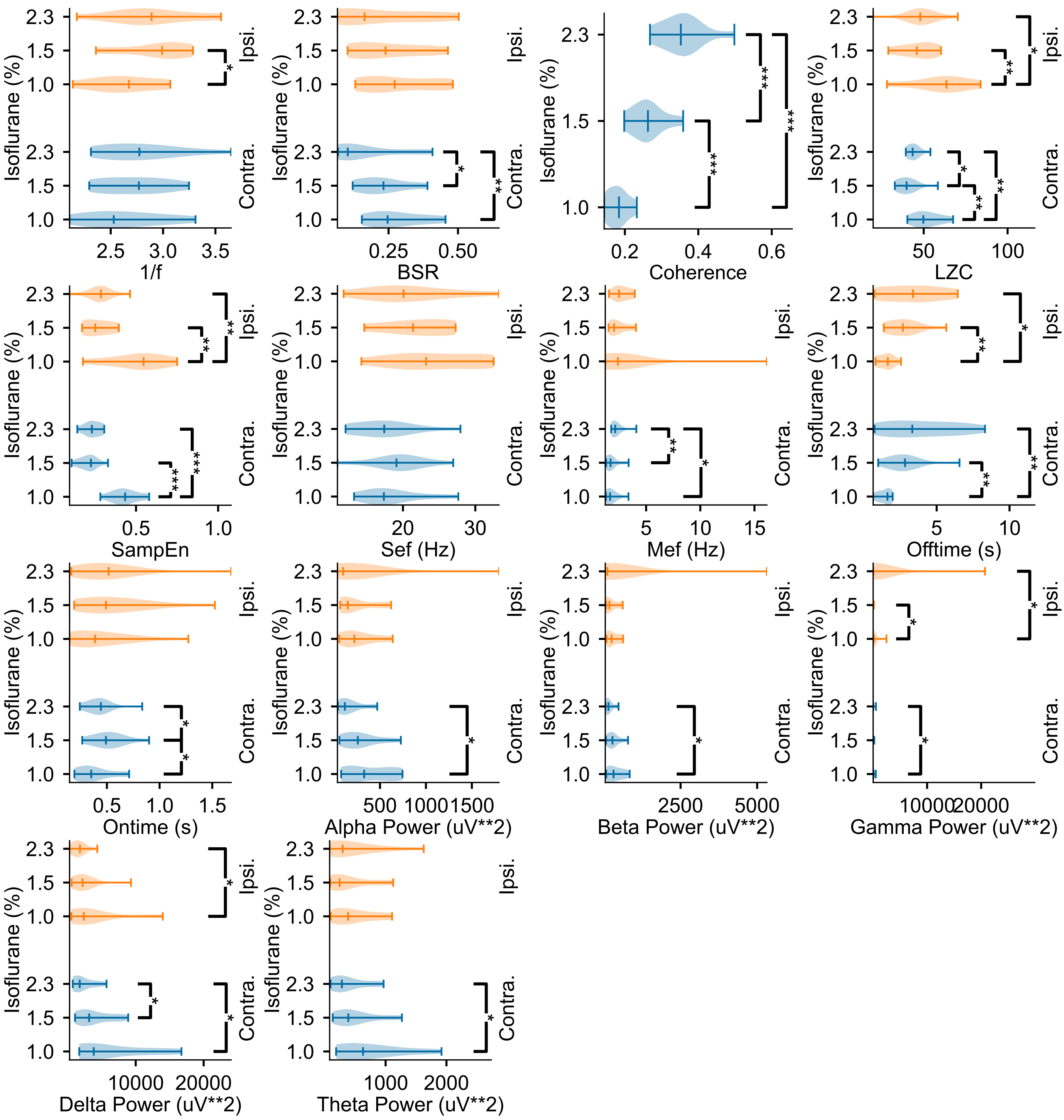

Figure S6. The modulation of all extracted features by isoflurane concentration. Significance testing computed with two-sided Mann-Whitney U test across isoflurane concentrations. Significant results are denoted with $*p < 0.05$, $**p < 0.01$, $***p < 0.01$

Table S1. P-Values of the two-sided Mann-Whitney-U test between the feature mean distributions in different isoflurane concentrations. $n = 11$. Feature means with $p < 0.05$ are listed in bold.

Feature	Channel<br>Isoflurane<br>Isoflurane	Contralat.<br>1.5	 2.3	Ipsilat.<br>1.5	 2.3
Coherence	1.0	**1.52×10^{-4}**	**4.08×10^{-5}**	**1.52×10^{-4}**	**4.08×10^{-5}**
	1.5		**6.46×10^{-4}**		**6.46×10^{-4}**
SampEn	1.0	**5.35×10^{-5}**	**9.10×10^{-5}**	**1.56×10^{-3}**	**2.91×10^{-3}**
	1.5		1.47×10^{-1}		3.96×10^{-1}
Offtime	1.0	**1.01×10^{-3}**	**9.04×10^{-3}**	**4.31×10^{-3}**	**2.44×10^{-2}**
	1.5		1.79×10^{-1}		2.56×10^{-1}
LZC	1.0	**1.93×10^{-3}**	**4.31×10^{-3}**	**6.29×10^{-3}**	**1.28×10^{-2}**
	1.5		**3.81×10^{-2}**		4.48×10^{-1}
Mef	1.0	3.96×10^{-1}	**2.09×10^{-2}**	4.22×10^{-1}	1.79×10^{-1}
	1.5		**6.29×10^{-3}**		1.19×10^{-1}
BSR	1.0	1.06×10^{-1}	**9.04×10^{-3}**	1.62×10^{-1}	1.06×10^{-1}
	1.5		**3.81×10^{-2}**		2.35×10^{-1}
Delta Power	1.0	1.97×10^{-1}	**1.28×10^{-2}**	2.56×10^{-1}	**3.81×10^{-2}**
	1.5		**2.84×10^{-2}**		1.32×10^{-1}
Total Power	1.0	1.79×10^{-1}	**1.78×10^{-2}**	1.97×10^{-1}	8.40×10^{-2}
	1.5		**3.30×10^{-2}**		2.35×10^{-1}
Alpha Power	1.0	2.35×10^{-1}	**1.78×10^{-2}**	3.00×10^{-1}	2.15×10^{-1}
	1.5		9.45×10^{-2}		2.77×10^{-1}
Beta Power	1.0	2.56×10^{-1}	**1.78×10^{-2}**	1.79×10^{-1}	7.43×10^{-2}
	1.5		8.40×10^{-2}		2.15×10^{-1}
Gamma Power	1.0	1.32×10^{-1}	**3.81×10^{-2}**	**2.09×10^{-2}**	**4.39×10^{-2}**
	1.5		1.79×10^{-1}		3.47×10^{-1}
Ontime	1.0	**4.39×10^{-2}**	1.47×10^{-1}	1.32×10^{-1}	2.56×10^{-1}
	1.5		**2.44×10^{-2}**		3.47×10^{-1}
1/f	1.0	3.00×10^{-1}	2.15×10^{-1}	**2.84×10^{-2}**	5.75×10^{-2}
	1.5		3.47×10^{-1}		2.77×10^{-1}
Theta Power	1.0	1.62×10^{-1}	**3.30×10^{-2}**	2.35×10^{-1}	2.77×10^{-1}
	1.5		1.79×10^{-1}		5.00×10^{-1}
Sef	1.0	1.79×10^{-1}	3.23×10^{-1}	1.79×10^{-1}	9.45×10^{-2}
	1.5		8.40×10^{-2}		2.35×10^{-1}

Table S2. Median values of all features, over all $n = 11$ mice.

	1.0	1.5	2.3
Contralateral 1/f	2.66	2.76	2.81
Ipsilateral 1/f	2.67	2.95	2.88
Coherence	0.17	0.25	0.35
Contralateral BSR	0.25	0.23	0.12
Ipsilateral BSR	0.26	0.25	0.13
Contralateral LZC	52.00	40.00	45.00
Ipsilateral LZC	64.00	46.00	50.00
Contralateral Mef	1.59 Hz	1.70 Hz	1.99 Hz
Ipsilateral Mef	1.97 Hz	1.91 Hz	2.58 Hz
Contralateral Offtime	1.40 s	2.54 s	3.52 s
Ipsilateral Offtime	1.34 s	2.41 s	3.31 s
Contralateral Ontime	0.32 s	0.46 s	0.37 s
Ipsilateral Ontime	0.31 s	0.43 s	0.38 s
Contralateral SampEn	0.42	0.20	0.23
Ipsilateral SampEn	0.53	0.26	0.29
Contralateral Sef	17.73 Hz	19.24 Hz	16.51 Hz
Ipsilateral Sef	23.41 Hz	21.75 Hz	19.64 Hz
Contralateral Alpha Power	232.23 μV^2	250.67 μV^2	112.27 μV^2
Ipsilateral Alpha Power	160.18 μV^2	159.28 μV^2	81.44 μV^2
Contralateral Beta Power	239.70 μV^2	266.35 μV^2	102.10 μV^2
Ipsilateral Beta Power	169.88 μV^2	169.94 μV^2	73.34 μV^2
Contralateral Gamma Power	73.15 μV^2	63.51 μV^2	25.40 μV^2
Ipsilateral Gamma Power	65.66 μV^2	47.92 μV^2	24.36 μV^2
Contralateral Delta Power	3735.47 μV^2	2799.92 μV^2	1524.08 μV^2
Ipsilateral Delta Power	2172.46 μV^2	1848.03 μV^2	960.36 μV^2
Contralateral Theta Power	466.57 μV^2	415.59 μV^2	318.52 μV^2
Ipsilateral Theta Power	328.25 μV^2	291.60 μV^2	197.21 μV^2

Table S3. P-Values of the Wilcoxon signed-rank tests performed testing features for hysteresis. Features with hysteresis $p < 0.05$ are listed in bold.

Channel Feature	Contralat.	Ipsilat.
Sef	1.75×10^{-1}	$\mathbf{9.77 \times 10^{-4}}$
Gamma Power	2.40×10^{-1}	$\mathbf{9.77 \times 10^{-4}}$
Beta Power	4.13×10^{-1}	$\mathbf{1.95 \times 10^{-3}}$
Mef	4.13×10^{-1}	$\mathbf{6.84 \times 10^{-3}}$
Total Power	2.40×10^{-1}	$\mathbf{6.84 \times 10^{-3}}$
Delta Power	8.31×10^{-1}	$\mathbf{6.84 \times 10^{-3}}$
BSR	5.77×10^{-1}	$\mathbf{9.77 \times 10^{-3}}$
Alpha Power	1.00	$\mathbf{9.77 \times 10^{-3}}$
LZC	8.98×10^{-1}	$\mathbf{1.37 \times 10^{-2}}$
1/f	1.47×10^{-1}	$\mathbf{1.86 \times 10^{-2}}$
Ontime	7.65×10^{-1}	$\mathbf{2.44 \times 10^{-2}}$
SampEn	8.98×10^{-1}	5.37×10^{-2}
Theta Power	1.75×10^{-1}	1.75×10^{-1}
Offtime	5.77×10^{-1}	4.13×10^{-1}
Coherence	8.31×10^{-1}	8.31×10^{-1}

Table S4. Median values of relative hysteresis differences.

	Contralateral	Ipsilateral
Beta Coherence	−0.008	−0.008
Coherence	0.008	0.008
1/f	0.014	0.043
LZC	0.019	−0.133
Delta Power	0.022	0.132
BSR	0.036	0.164
Ontime	−0.057	0.435
Mef	−0.059	−0.387
Alpha Power	−0.060	−0.398
Sef	−0.069	−0.212
Offtime	0.173	0.086
Alpha Coherence	0.096	0.096
Theta Power	0.176	−0.103
Gamma Power	−0.104	−0.423
Beta Power	−0.107	−0.398
SampEn	0.118	−0.273
Delta Coherence	0.144	0.144
Theta Coherence	0.156	0.156
Total Power	0.156	0.928
Gamma Coherence	−0.169	−0.169

REFERENCES

Hu, J., Gao, J., and Principe, J. C. (2006). Analysis of biomedical signals by the lempel-ziv complexity: the effect of finite data size. *IEEE Transactions on Biomedical Engineering* 53, 2606–2609

Richman, J. S. and Moorman, J. R. (2000). Physiological time-series analysis using approximate entropy and sample entropy. *American Journal of Physiology-Heart and Circulatory Physiology* 278, H2039–H2049
